# Ligand-Tuned CISS-Effect of Atomically Precise Metal Oxido Nanoclusters

**DOI:** 10.1021/acs.jpclett.6c01759

**Published:** 2026-07-06

**Authors:** D. Hornig, T. N. H. Nguyen, L. T. Baczewski, M. Gruschwitz, A. Undisz, M. Mehring, C. Tegenkamp

**Affiliations:** † Faculty of Natural Sciences, Institute of Chemistry, Coordination Chemistry, 38869 Chemnitz University of Technology, Chemnitz 09107, Germany; ‡ Institute of Physics, 38869 Chemnitz University of Technology, Chemnitz 09107, Germany; ¶ Institute of Physics, Polish Academy of Sciences, Warszawa 02-668, Poland; ∥ Institute of Materials Science and Engineering, 38869 Chemnitz University of Technology, 09125 Chemnitz, Germany; ⊥ Center of Materials, Architectures and Integration of Nanomembranes, 38869 Chemnitz University of Technology, Chemnitz 09126, Germany; # TEM-Center, 38869 Chemnitz University of Technology, 09125 Chemnitz, Germany

## Abstract

Chirality-induced spin effects, first observed in hybrid interface structures, are distinguished by their remarkable robustness. For practical applications and tunability, chemically modular design concepts are particularly promising. Here, we demonstrate chirality-induced spin selectivity (CISS) in sub-3 nm metal oxido nanoclusters, using bismuth-based systems as a model platform that can be selectively activated by chiral carboxylate ligands. While both chiral and achiral nanoclusters adsorb similarly onto magnetic Au/Co/Au nanostructures via sulfur–metal bonding, the adsorption process alone does not induce a CISS effect, i.e., not imparting chirality to the interface. Our findings establish a modular molecular design strategy that enables the targeted functionalization of chiral metal oxido nanoclusters, opening perspectives for their integration into spin-dependent applications, including optoelectronic systems.

## Graphical abstract

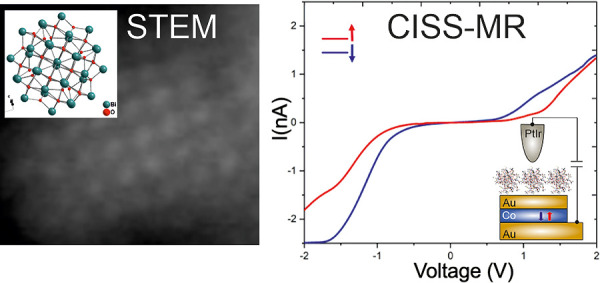

The chiral-induced spin selectivity (CISS) effect is a fascinating phenomenon that continues to pose a significant conceptual challenge.^1^ It is particularly intriguing because it bridges fundamental questions in spin chemistry and physics with potential technological applications such as in spintronics, asymmetric catalysis, or photoelectrochemical water splitting.^2,3^ However, despite the numerous experiments, a comprehensive theoretical framework that fully accounts for the observed magnitude and robustness of the effect is still lacking.^4,5^

Over the past nearly two decades, a wide range of experiments have demonstrated that spin polarization generated through molecular chirality is a remarkably robust effect. This behavior has been observed in numerous systems, most prominently in molecular systems, and has been reported even under ambient conditions.

A large number of the early experiments were performed using polypeptide-based systems.^6–8^ These helical molecular structures played an important role in shaping the initial interpretation of the CISS effect, suggesting that it is primarily an intramolecular phenomenon, where the chiral molecule itself acts as a spin polarizer during electron transport.^9^

However, later experiments on chiral but nonmolecular monolayers clearly indicated that the situation is more complex. These studies demonstrated that the interface between the chiral layer and the metallic electrode can play a crucial role in the emergence of spin polarization.^10^ Moreover, even achiral species were found to induce a CISS effect after adsorption.^11^ This view is further supported by various so-called “spinterface” models, which emphasize the importance of interfacial electronic structure and spin-dependent hybridization in generating the observed spin selectivity^12–16^

Due to the robustness of the CISS effect, applications and further developments are readily conceivable.^17^ For instance, chiral linkers in combination with donor–acceptor systems have been used as a model system to tune radical pair spin dynamics for future qubit applications.^18^ Other applications include the modification of surface magnetism^19^ or light-induced effects.^20^ Moreover, extended molecular assemblies are currently under investigation, and the development of chiral metal–organic framework systems appears promising for chiral electronic applications.^21–23^

Based on this background, modular chemical concepts are highly valuable, as they allow the influence of the molecular system to be tuned and investigated, both in solution and when adsorbed on substrate surfaces. One promising approach is the use of chiral metal oxido clusters of main group metals, which recently have attracted great attention for their CPL (circularly polarized luminescence) activity, second-order nonlinear optics (NLO), and drug delivery (DD).^24,25^ CPL has widespread potential applications in encrypted information transmission and storage, chiroptical materials, and bioencoding. Moreover, they are cost-effective, environmental benign and show flexibility in size and physical properties. Studies of their chiral properties, especially with reference to atomically precise chiral coinage metal nanoclusters, are still remaining unexplored.^26–29^

Among the metal oxido nanoclusters of main group metals reported so far, those of bismuth offer the largest diversity with regard to cluster size and ligand shell, while being environmentally benign.^30–32^ These atomically precise metal oxido nanoclusters offers a possibility of optical properties tuning through doping of the metal oxido core and ligand shell modification, both without change of the size of the metal oxido cluster core as was demonstrated recently for bismuth oxido nanoclusters (BiO-NC) of the type [Bi_38_O_45_(ligand)_24_].^33–35^ Thus, these sub 3 nm nanoclusters bridge the gap between small molecules and nanoparticles and provide a platform to study ligand driven spin polarization effects of small nanoobjects. Proper ligand choice was demonstrated to allow for the controlled deposition of such functionalized BiO-NCs on different substrates such as HOPG and Au.^36,37^

In this work, we report for the first time on the CISS effect present in chiral metal oxido nanoclusters that builds a bridge between molecular and extended-structure-driven effects. Through chemical functionalization, achiral 3-methylthiopropionate- and chiral Boc-d- and Boc-l-methionine-functionalized BiO-NCs were assembled as monolayers from a solution onto epitaxial magnetic Au/Co/Au nanostructures. The robustness of BiO-NC structures was confirmed by electrospray ionization mass spectrometry (ESI-MS), dynamic light scattering (DLS), nuclear magnetic resonance (NMR), infrared (IR), and ultraviolet–visible (UV–vis) spectroscopy, as well as scanning tunneling microscopy (STM), while their chiral and spinselective properties (CISS effect) were analyzed using circular dichroism (CD) spectroscopy and scanning tunneling spectroscopy (STS). Anchoring on gold is provided by methylsufanyl binding in all cases, but it is demonstrated that chiral-induced spin selectivity was triggered by the enantiomeric molecular system.

Two bismuth oxido nanoclusters (BiO-NC) with opposite chirality, [Bi_38_O_45_(Boc-l-Met-O)_24_] and [Bi_38_O_45_(Boc-d-Met-O)_24_], together with a similar achiral nanocluster [Bi_38_O_45_(3-MTP-O)_24_] were synthesized via ligand exchange from $\left[{Bi}_{38}O_{45}{\left({NO}_{3}\right)}_{20}{\left(dmso\right)}_{28}\right]{\left({NO}_{3}\right)}_{4}\cdot 4dmso$ , allowing complete replacement of nitrate ligands by chiral or achiral organic carboxylates.^38^ The three different compounds with the chiral Boc-l-Met-O, Boc-d-Met-O and achiral 3-MTP-O ligands are denoted by l-BiO-NC, d-BiO-NC and A-BiO-NC, respectively (see also Figure 1(a)). Further details about the synthesis and cluster characterization are reported in the Supporting Information.

**Figure 1:**
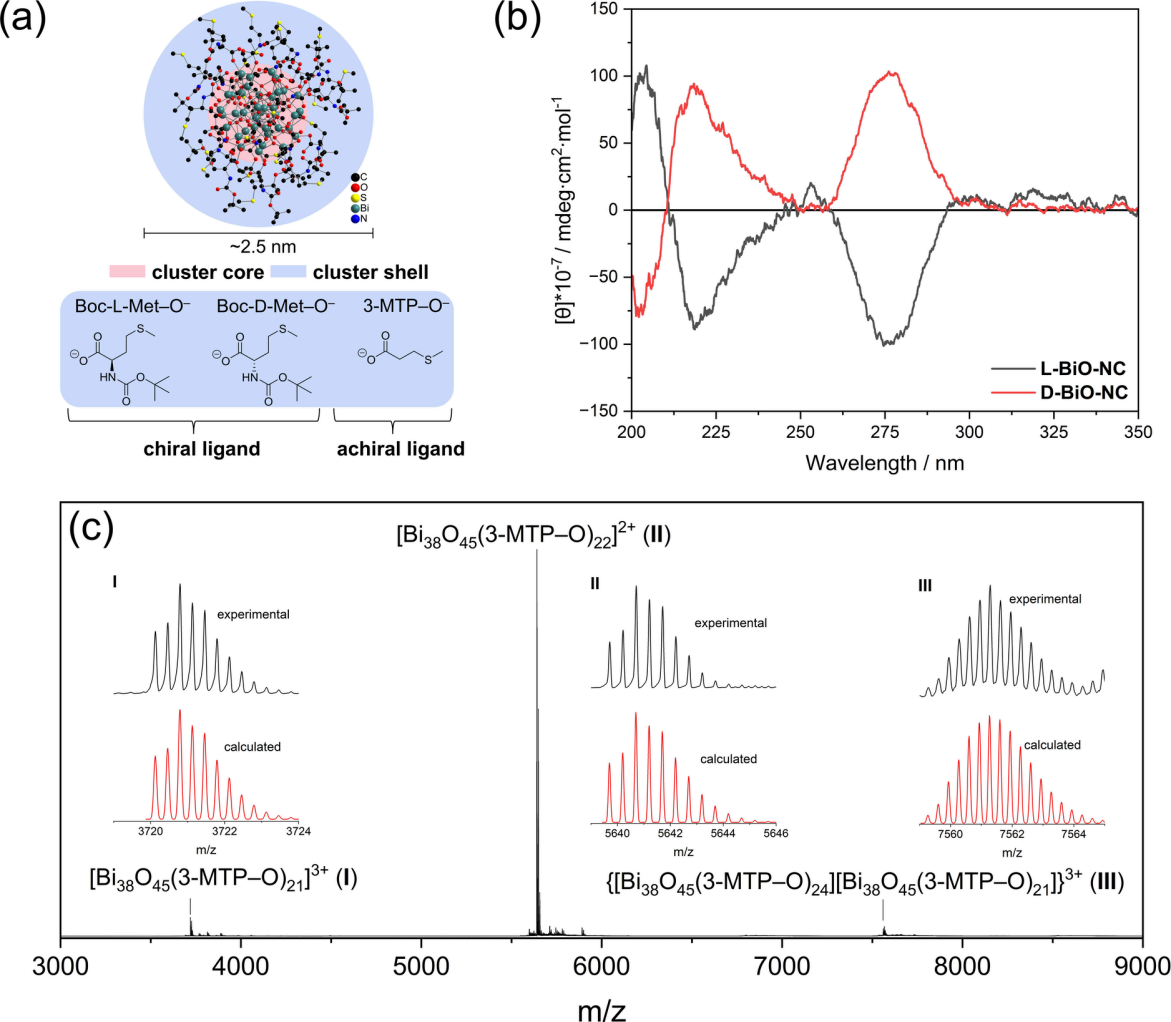
(a) Schematic molecular structure exemplarily given for [Bi_38_O_45_(Boc-l-Met-O)_24_] (l-BiO-NC). The lower part shows the three ligands used to provide two chiral NCs with opposite handeness and one achiral BiO-NC. (b) Circular dichroism (CD) measurements for l-BiO-NC and d-BiO-NC in EtOH (*c* = 3 · 10^–5^ mol L^–1^), demonstrating chiral activity. The spectrum for achiral A-BiO-NC is shown in the Supporting Information (Figure S12). (c) ESI mass spectrum for A-BiO-NC electrosprayed from a DMSO solution.

The functionalized BiO-NCs were deposited by drop-casting (from either an EtOH or a DMSO/EtOH solution, 1.5 mM) on molecular beam epitaxy grown magnetic nanostructures consisting of Al_2_O_3_/Pt/Au­(20 nm)/Co­(1.2 nm)/Au­(5 nm). The Co layer exhibits an out-of-plane anisotropy with a coercive field of 16 mT, which is easily switchable by an external magnetic field. Further details of the magnetic nanostructure are outlined elsewhere.^8,39^

The CISS-MR measurements were performed by using ambient scanning tunneling microscopy (STM) and spectroscopy (STS). STM tips were made from 0.25 mm of PtIr wire, and all measurements were carried out at 300 K. To quantify the spin polarization of transmitted electrons, I–V spectra were recorded at the set points *V*_*b*_ = 0.5 V and *I*_*t*_ = 100 pA. Each spectrum represents the average of at least ten measurements.

To study the CISS response in nanoscaled systems as a function of ligand effects, well-defined, highly pure, and thus monodisperse nanoobjects are required. Therefore, the sub 3 nm chiral metal oxido clusters l- and d-BiO-NCs and achiral A-BiO-NC were synthesized (Figure 1(a)).

Their composition and structural integrity were investigated in the solid state, in solution and in the gas phase. Powder X-ray diffraction confirmed the preservation of the {Bi_38_O_45_} cluster core after ligand exchange. The diffractograms show the characteristic reflections of amino acid functionalized bismuth oxido nanoclusters, with one intense reflection and a second significantly weaker one at 2θ < 10° (see Supporting Information). Preservation of cluster integrity in solution was further assessed by dynamic light scattering (DLS) measurements in solution. Hydrodynamic radii of 2.3–2.6 nm were determined for l- and d-BiO-NCs in ethanol and dimethyl sulfoxide (DMSO), while A-BiO-NC showed a smaller radius of 1.7 nm in DMSO, also consistent with a shift to higher angles in the PXRD pattern. The average Bi–Bi distance in the BiO cluster is around 3.7 ± 0.1 Å as revealed by small-angle X-ray scattering^40^ and recent transmission electron microscopy (STEM) measurements (see Supporting Information). Together with NMR data, these results confirm the stability of the nanoclusters.

To further assess the molecular composition of the clusters, ESI-MS measurements were carried out. In contrast to nanoparticles and quantum dots, the number of ligands is precisely defined by the polycationic cluster core [Bi_38_O_45_]^24+^ in combination with 24 monoanionic ligands. Accordingly, the ESI-mass spectra, shown exemplarily for A-BiO-NC in Figure 1(c), give typical doubly and triply charged BiO-NC cations (**I**, **II**) generated by loss of the appropriate number of counteranions. The spectrum is dominated by the mass peaks assigned to [Bi_38_O_45_(3-MTP-O)_22_]^2+^ (**II**) at *m*/*z* = 5640.7070. Noteworthy, a mass peak cluster assigned to a dimeric cluster aggregate was detected at high *m*/*z* = 7561.2821, indicating the formation of the triply charged species {[Bi_38_O_45_(3-MTP-O)_24_]­[Bi_38_O_45_(3-MTP-O)_21_]^3+^} (**III**) as a result of gas phase aggregation of a cationic cluster with a neutral one. Evidence for such larger aggregates in solution is not observed, demonstrating that this dimeric species is generated during electrospray ionization mass spectrometry. Circular dichroism (CD) measurements, shown in Figure 1(b), demonstrate the transfer of chiral information from enantiopure carboxylates of Boc-protected methionine to the bismuth oxido framework, enabling clear differentiation between the enantiomeric nanoclusters l-BiO-NC and d-BiO-NC. The spectra show a pronounced Cotton effect attributed to the ligand centered transition in the range of 200–240 nm. Besides this characteristic amino acid signal, a broader Cotton effect centered at approximately 275 nm is observed and is assigned to a ligand induced charge transfer effect. This bands appear as mirror-image signals for the nanoclusters functionalized with Boc-protected l- and d-methionine ligands and confirm the transfer of chiral information, thus resulting in enantiomeric nanoobjects. The achiral cluster A-BiO-NC does not show any signal in the CD spectrum (see Supporting Information). The chiral and achiral organic carboxylates were selected to introduce methylsulfanyl end groups, allowing stable anchoring of the nanoclusters on gold surfaces and enabling their deposition as self-assembled monolayers (SAMs) on Au/Co/Au magnetic substrates.

As a substrate for adsorption and the CISS measurements, we employed Au/Co/Au nanostructures. Thereby, the top Au layer, with a thickness of 5 nm, enables the use of well-established surface chemistry, in this case, the adsorption of the nanoclusters onto the substrate. The buried Co layer exhibits an out-of-plane magnetization, which can be switched by an external magnetic field, allowing the quantification of spin transport (see below).

A consequence of the heterostructure grown by MBE is that the surfaces are slightly rougher compared to single crystals but, as can be seen in inset in Figure 2(a), typical terrace widths in the range of 10–20 nm are present, what is sufficient for the adsorption and spectroscopic measurements performed here.^39^ In panels (a–c), the cluster structures of the three nanoclusters (NCs) are clearly resolved after adsorption from solution. In all experiments, either DMSO or a DMSO/EtOH mixture were used, resulting in densely packed cluster arrangements in each case. The observed homogeneity of the films suggest that the thiosulfanyl-terminated ligand shells effectively anchor the clusters to the Au/Co/Au surface, stabilizing the monolayer.

**Figure 2:**
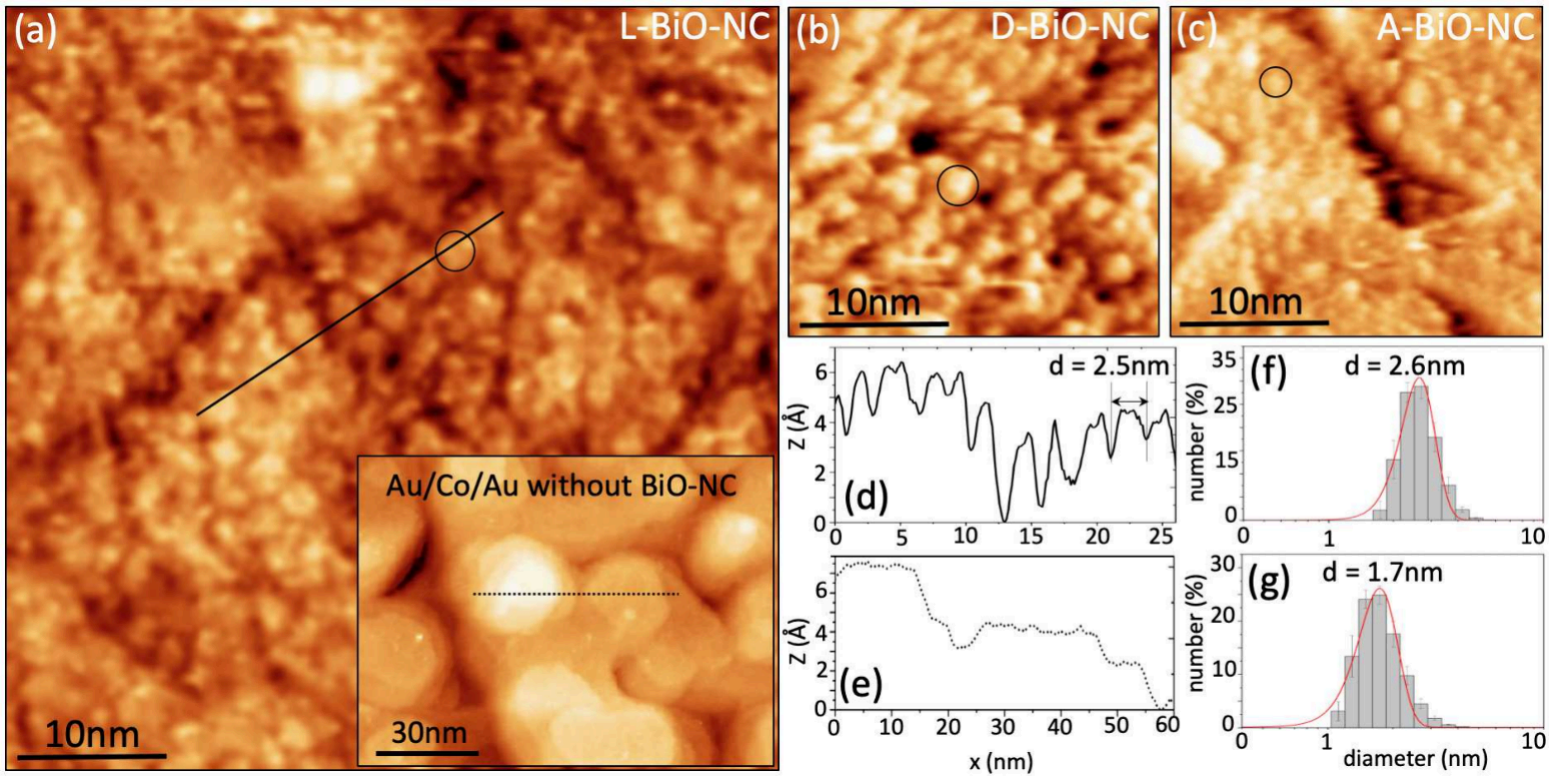
STM images of l-BiO-NC (a), d-BiO-NC (b) and the A-BiO-NC (c) clusters adsorbed from solution on Au/Co/Au surface. The inset in (a) shows the bare clean Au layer surface of the Au/Co/Au nanostructure before deposition. (d,e) Apparent heights of l-BiO-NC film and Au/Co/Au taken on figure a (solid and dotted black lines, respectively). The height profiles for the two other nanoclusters are shown in the Supporting Information. (f,g) Results of dynamic light scattering experiments (DLS) of d-BiO-NC in ethanol and of A-BiO-NC in DMSO. The results for l-BiO-NC are similar to to those shown in panel (f). The influence of the solvent on the hydrodynamic diameter and CISS-effect is negligible (see Supporting Information).

The cluster sizes determined from the STM topography images and corresponding line scans (e.g., as shown in Figure 2(d)) reveal average diameters of approximately 2.5 ± 0.2 nm for l-BiO-NC and d-BiO-NC and 1.9 ± 0.2 nm for A-BiO-NC (see also Supporting Information). These values are in very good agreement with the average sizes obtained by DLS in solution (Figure 2(f,g)). This indicates that the clusters remain intact upon deposition on the surface, consistent with our previous assumption.^37^ The structural properties of the cluster films appear similar; thus, the different ligands do not significantly alter the adsorption or aggregation behavior on the surface and in the liquid phase, respectively. The formation of larger aggregates in the liquid phase, as observed for polypeptides, does not occur.^41^

To demonstrate chirality-induced spin polarization due to the clusters, STS measurements were performed as a function of the magnetization orientation of the Co layer. It has been shown in our previous work, as well as by others, that spin polarization can be probed by STS.^8,10,39,41^ In this approach, the spin polarization induced by the CISS effect is detected via the magnetic Co layer, effectively acting as a spin valve. The difference in transmission for the two opposite magnetization directions thus provides a direct measure of the CISS effect and the so-called CISS-MR value is given by CISS-MR ∝ *I*_*↑*_ – *I*_*↓*_. Reference measurements without clusters were performed in advance in order rule out parasitic effects.

In Figure 3 the IV curves obtained after deposition of the different types of clusters are given. Despite the structural similarity, STS measurements show a clear inversion of the I–V characteristics upon changing the chirality of the ligands, as shown in Figure 3(a) and (b). Specifically, the IV-curves of the enantiomeric l-BiO-NC and d-BiO-NC exhibit opposite behavior of the electron transmissions as a function of the magnetization direction of the Co film, demonstrating a strong chiral-induced spin selectivity, consistent with previously reported CISS effects in chiral peptides. Quantitative analysis of these IV curves indicates that the CISS-MR in the chiral films reaches approximately 45–50% which is in the same range as reported for polypeptides.^7,41^

**Figure 3:**
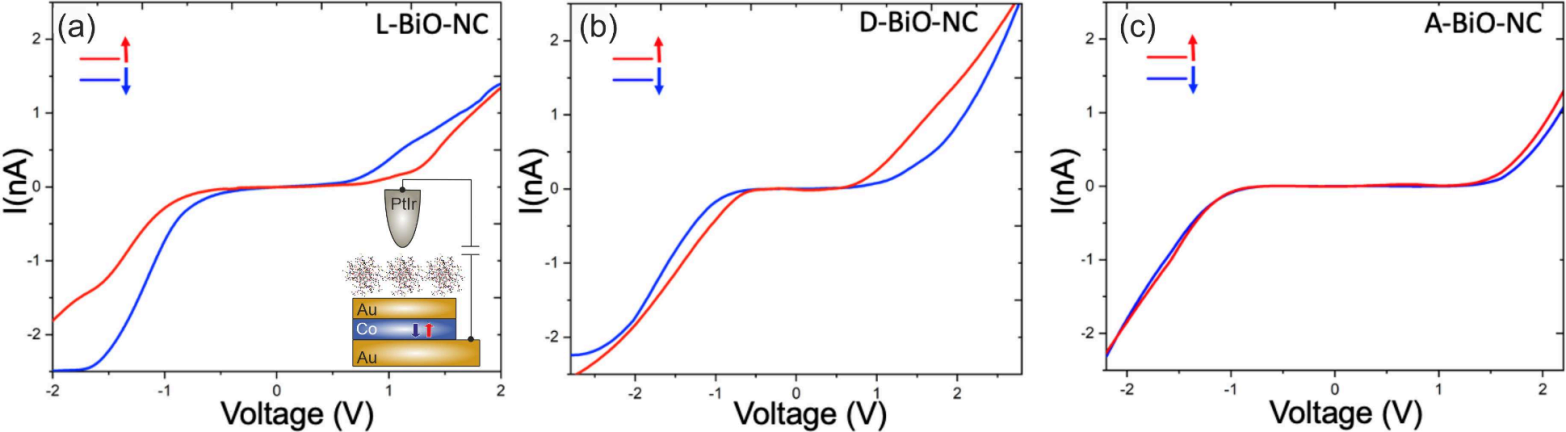
CISS I–V curves for l-BiO-NC (a), d-BiO-NC (b), and A-BiO-NC (c) on Au/Co/Au substrates. A clear difference in the I–V characteristics depending on the substrate magnetization is observed only for BiO-NCs with chiral ligands in (a) and (b). All measurements were performed at 300 K with a set point of +0.5 V and 100 pA. The inset in panel a shows the schematic setup for the STS measurements for different out-of-plane magnetizations of the Co-layer (blue, red).

In order to prove that the effect is not based on the interaction of the nanoclusters with the gold surface, the achiral A-BiO-NC films were investigated. These experiments revealed nearly identical IV curves for both magnetization directions (Figure 3(c)), indicating no CISS effect. This is remarkable because all bismuth oxido nanoclusters share an identical {Bi_38_O_45_} core as well as the same anchor group and an equal number of ligands at their metal oxido surface. The most crucial change is the difference in chirality of the ligands which correlates nicely with the magnitude and sign of the CISS effect. Also, the bonding via the methylsulfanyl in our case is not inducing an adsorption-induced chirality, which in turn also could result in a spin polarization.^11^

The apparent band gap deduced from Figure 3c is approximately 3 eV, in qualitative agreement with previous measurements and with the optical band gaps determined by UV–vis spectroscopy^37^ (see Supporting Information). Although the optical band gaps of all three clusters lie in the range of 3.5–3.6 eV, the I–V curves in Figure 3 reveal a smaller apparent band gap for the clusters. We attribute this discrepancy to the superposition of a background signal arising from the CISS effect probed in the spin-valve measurements. Consequently, the intrinsic band gap of the BiO-NCs cannot be directly extracted from the I–V characteristics.

As mentioned, the cluster core is surrounded by 24 ligands, resulting in a structure that microscopically resembles a hedgehog-like geometry, at least in solution. Assuming that this configuration is preserved upon adsorption, the tunneling process effectively occurs through the two ligands and the central core. Although both ligands possess the same chirality, they are, in this idealized scenario, rotated by π with respect to each other. In our recent work on polypeptides, we have shown that the CISS signal reverses upon rotation of the helical unit.^39^ This behavior has also been confirmed by break-junction experiments.^42^ Under the assumption that the chiral linker groups act as spin polarizers, one would therefore expect the CISS effect to cancel out. The fact that a finite effect is nevertheless observed likely supports a model in which the hybrid interface between a molecular system and a metal surface plays a central role. The dependence of the transmission on the magnetization of the Co layer for the chiral BiO-NCs is identical to that observed for polypeptides adsorbed on the same Au/Co/Au nanostructure.^41^ At the same time, we cannot exclude that the interface between the bismuth oxido core and the chiral organic shell also contributes to the spin polarization and may further modify the spin polarization at the metal–molecule interface.

In conclusion, we have shown on the example of atomically precise metal oxido nanoclusters of bismuth (BiO-NCs) that the CISS effect can be tuned in molecular spherical nanoobjects by the nature of the ligand. We used an amino acid–based ligand to induce the chirality and methylsulfanyl groups to ensure strong chemical bonding to gold while preserving the modular design concept. By their molecular nature the nanoclusters provide a monodisperse platform, chemical flexibility with regard to the choice of ligands and tunable optical properties, e.g. by doping, as reported elsewhere. Here we demonstrate that chirality of a nanoobject in close proximity to a metal contact induces the CISS effect, but without inducing surface chirality and without showing a directional chiral axis. These findings support the concept of a spin-active interface (“spinterface”). Future experiments on doped bismuth oxido nanoclusters will reveal to what extent photoinduced CISS interfaces can be realized, potentially enabling applications in optospintronics.

## Supplementary Material



## Notes

Materials and methods, synthesis of the BiO clusters, characterization by NMR, ESI-MS, ATR-IR, CD UV–vis, STEM, and IV-spectroscopy (PDF)
