# Supplementary material for: Ligand-Tuned CISS-Effect of Atomically Precise Metal Oxido Nanoclusters
Source: J Phys Chem Lett. 2026 Jul 6;17(28):7922–7. doi: 10.1021/acs.jpclett.6c01759 (PMC13383834; doi:10.1021/acs.jpclett.6c01759)
Supplement: Supplementary file 1 [file jz6c01759_si_001.pdf]

Supporting Information: Ligand-tuned CISS-effect of atomically precise metal oxido nanoclusters

D. Hornig,[†,⊥] T.N.H. Nguyen,[‡] L.T. Baczewski,[¶] M. Gruschwitz,[‡,#] A. Undisz,[§,#] M. Mehring,[*,†,⊥] and C. Tegenkamp[*,‖,#]

†*Faculty of Natural Sciences, Institute of Chemistry, Coordination Chemistry, Chemnitz University of Technology, Chemnitz 09107, Germany*

‡*Institute of Physics, Chemnitz University of Technology, Chemnitz 09107, Germany*

¶*Institute of Physics, Polish Academy of Sciences, Warszawa 02-668, Poland*

§*Institute of Materials Science and Engineering, Chemnitz University of Technology, 09125 Chemnitz, Germany*

‖*Institute of Physics, Chemnitz University of Technology, Chemnitz 09107 Germany*

⊥*Center of Materials, Architectures and Integration of Nanomembranes, Chemnitz University of Technology, Chemnitz 09126, Germany*

#*TEM-Center, Chemnitz University of Technology, 09125 Chemnitz, Germany*

E-mail: michael.mehring@chemie.tu-chemnitz.de; christoph.tegenkamp@physik.tu-chemnitz.de

In the following, additional experimental details and characterization data are provided to complement the results presented in the main manuscript. Specifically, this includes:

- Detailed materials and methods for the synthesis of the bismuth oxido nanoclusters (BiO-NCs)

- Comprehensive characterization of the BiO-NCs by NMR, ESI-MS, ATR-IR, CD, PXRD, DLS, and UV–vis spectroscopy
- STEM data
- Additional I–V spectra from CISS measurements

1. Materials and Methods

N-(*tert*-butoxycarbonyl)-L-methionine (99%), *N*-(*tert*-butoxycarbonyl)-D-methionine (98%) and Na_2CO_3 (99.5%) were purchased from Sigma-Aldrich, while 3-(Methylthio)propionic acid (98%) was obtained from BLD Pharm. $Bi(NO_3)_3 \cdot 5\,H_2O$ (98%), spectroscopic grade ethanol (99.9%) was purchased from Alpha Aesar and dimethyl sulfoxide (99.9%) from Fisher Chemicals. All chemicals were used without further purification.
The synthesis of $[Bi_{38}O_{45}(NO_3)_{20}(dmso)_{28}](NO_3)_4 \cdot 4\,dmso$ (A),[1] Boc-L-Met−ONa (B),[2] and $[Bi_{38}O_{45}$(Boc-L-Met−O)$_{24}]$ (L-BiO-NC)[2] was carried out according to the procedure described in the literature. The sodium salts Boc-D-Met−ONa (C) and sodium 3-methylthiopropionate (D), as well as the nanocluster $[Bi_{38}O_{45}$(Boc-D-Met−O)$_{24}]$ (D-BiO-NC) and $[Bi_{38}O_{45}$(3-MTP−O)$_{24}]$ (A-BiO-NC) were synthesized in accordance with the synthesis procedure of compounds B and L-BiO-NC.[2]

Synthesis of $[Bi_{38}O_{45}$(Boc-D-Met−O)$_{24}]$ (D-BiO-NC)

$[Bi_{38}O_{45}(NO_3)_{20}(dmso)_{28}](NO_3)_4 \cdot 4\,dmso$ (A, 500 mg, 0.040 mmol) was dissolved in 25 mL dmso under stirring at 80 °C for 1 h to give a colorless solution. Boc-D-Met−ONa (C, m = 394 mg, n = 1.45 mmol) was added to the colorless solution and the mixture was kept at 80 °C for 4 h. The hot solution was subsequently filtered and solid D-BiO-NC was obtained after slow evaporation of the solvent over 3 weeks. The solid was washed with 20 mL of deionized water and then dried under vacuum at 60 °C for 2 h. Compound D-BiO-NC was

obtained as a colorless solid (436 mg, 0.030 mmol, $\eta = 75\%$ based on bismuth in A).

Elemental analysis CHNS (%), exp. and calcd. for $Bi_{38}O_{141}C_{240}H_{432}N_{24}S_{24}$ ($M = 14620.94$ g·mol^{-1}): C 19.11 (19.72); H 2.98 (2.98); N 2.56 (2.30); S 4.70 (5.28). ^{1}H NMR (500.30 MHz, dmso-d_6, 298 K): $\delta = 6.33$ (s, 1 H), 3.99 (s, 1 H), 3.33 (s, H_2O), 2.54 (s, dmso$_{\text{coord.}}$), 2.46 (m, 2 H), 2.10 (m, 1 H), 2.04 (s, 3 H), 1.88 (m, 1 H), 1.38 (s, 9 H). ^{13}C NMR (125.81 MHz, dmso-d_6, 300 K): $\delta = 178.02$, 154.99, 77.84, 54.41, 32.48, 30.19, 28.23, 14.95. ATR-IR (cm^{-1}): 3700–3100 w, 2974 w, 2915 w, 1683 s, 1559 s, 1494 s, 1441 w, 1388 s, 1364 s, 1246 m, 1160 s, 1048 s, 1022 s, 959 m, 859 m, 777 m, 760 m, 500 s, 461 s.

Synthesis of $[Bi_{38}O_{45}(3\text{-MTP}-O)_{24}]$ (A-BiO-NC)

$[Bi_{38}O_{45}(NO_3)_{20}(dmso)_{28}](NO_3)_4 \cdot 4\,dmso$ (A, 750 mg, 0.061 mmol) was dissolved in 30 mL dmso under stirring at 80 °C for 1 h to give a colorless solution. Sodium 3-methylthiopropionate (D, $m = 310$ mg, $n = 2.18$ mmol) was added to the colorless solution and the mixture was kept at 80 °C for 4 h. The hot solution was subsequently filtered, and a colorless solid was obtained after slow evaporation of the solvent over 3 weeks. Because the first ligand exchange was incomplete, i.e. nitrate ligands were not completely substituted as revealed by IR spectroscopy and ESI-MS, the isolated solid ($m = 631$ mg) was subjected to a second reaction under identical conditions with sodium 3-methylthiopropionate ($m = 261$ mg) in 20 mL dmso following the same work up procedure. After evaporation of the solvent, the solid was washed with 20 mL of deionized water and then dried under vacuum at 60 °C for 2 h. Compound A-BiO-NC was obtained as a colorless solid (351 mg, 0.030 mmol, $\eta = 50\%$ based on bismuth in A).

Elemental analysis CHNS (%), exp. and calcd. for $Bi_{38}O_{93}C_{96}H_{168}S_{24}$ ($M = 11521.12$ g·mol^{-1}): C 10.01 (10.01); H 1.54 (1.47); S 6.66 (6.68). ^{1}H NMR (500.30 MHz, dmso-d_6, 298 K): $\delta = 3.33$ (s, H_2O), 2.67 (t, 2 H), 2.54 (s, dmso$_{\text{coord.}}$), 2.41 (t, 2 H), 2.07 (s, 3 H).

^{13}C NMR (125.81 MHz, dmso-d_6, 300 K): δ = 178.21, 37.17, 30.20, 14.81. ATR-IR (cm^{-1}): 3700–3100 w, 2965 w, 2910 w, 2828 w, 1519 s, 1410 m, 1375 s, 1298 m, 1268 m, 1204 m, 1143 m, 1016 w, 984 m, 928 m, 839 m, 783 w, 760 w, 721 w, 669 m, 466 s.

Material Characterization

Powder X-ray diffractograms were measured at ambient temperature using a STOE *Stadi P* diffractometer (Darmstadt, Germany) equipped with Ge(111)-monochromatized Cu-K$_\alpha$ radiation (λ = 1.54056 Å, 40 kV, 40 mA). The full width at half maximum (FWHM) was corrected for instrumental broadening using a LaB_6 standard (SRM 660) purchased from the National Institute of Standards and Technology (NIST). The value of β was corrected according to $\beta^2 = \beta^2_{\text{measured}} - \beta^2_{\text{instrument}}$, where β_{measured} and $\beta_{\text{instrument}}$ are the FWHMs of the measured and standard profiles, respectively. ^{1}H and ^{13}C{^{1}H} NMR spectra were recorded at ambient temperature in DMSO-d_6 (dried over 4 Å molecular sieve) using a Bruker *Avance III 500* spectrometer operating at 500.30 and 125.81 MHz, respectively, and were referenced internally to the deuterated solvent relative to $Si(CH_3)_4$ (δ = 0.00 ppm). CHNS analyzes were performed with a Foss Heraeus *Vario EL* analyzer. Infrared spectra were recorded using a *Nicolet iS 5 FT-IR (Fourier-transform infrared spectroscopy)* spectrometer (Thermo Scientific) equipped with an *iD7 AR-coated diamond crystal ATR accessory.* Solid samples were pressed onto the crystal with 40 pounds. Spectra were recorded with 32 scans at a resolution of 4 cm^{-1} using Omnic 9 software. UV-vis spectroscopy was carried out using a Cary 60 UV-vis spectrometer (Agilent Technologies) equipped with a *Barrelino* ™ (Harrick Scientific Products) remote diffuse reflection probe, using $BaSO_4$ as reference. The particle size distribution (PSD) was determined using a *Zetasizer Nano ZS* (Malvern Instruments) by dynamic light scattering (DLS). Using laser light (λ = 633 nm, P = 4 mW) as light source, suspensions with particle sizes ranging from 0.4 nm to 6 μm can be investigated. Measurements were performed at a scattering angle of 173° (non-invasive backscatter, NIBS default setting). Powders were dissolved in ethanol or dmso (β = 20 g L^{-1}), filled into glass cuvettes

(DTS0012) and measured at $T = 20\,°C$. PSD calculation was performed automatically according to "Mie theory" assuming spherical particles. Circular dichroism (CD) spectra were recorded using a J-1500 circular dichroism spectrophotometer (Jasco Deutschland GmbH, Pfungstadt, Germany) equipped with a standard air-cooled 150 W xenon lamp as a light source. Measurements were carried out in quartz cuvettes with a path length of 1 mm over a wavelength range of 200–400 nm for D-BiO-NC and L-BiO-NCs and 240–400 nm for A-BiO-NC. The experimental parameters were as follows: scanning speed of 100 $nm\,min^{-1}$, a bandwidth of 1.00 nm, a data pitch of 0.1 nm, a data integration time (D.I.T.) of 2 s, and averaging over three scans. For CD measurements, D-BiO-NC and L-BiO-NC were dissolved in spectroscopic grade ethanol (99.9%, Alpha Aesar), whereas A-BiO-NC was dissolved in chloroform, each at a concentration of $10^{-3}\,mol\,L^{-1}$. Electrospray ionization mass spectrometry (ESI-MS) was carried out using a Bruker Trapped Ion Mobility Spectrometry (tims) time-of-flight (TOF) mass spectrometer (Bruker Daltonik GmbH, Bremen, Germany) with a mass accuracy of < 0.8 ppm (mass drift over 8 hours with $\Delta T < 1$ K: < 2 ppm) and a mass resolution of 50 000 FSR (full sensitivity resolution at $m/z = 1221$). Calibration was done in the m/z range of 100–10 000 using cesium perfluoroheptonate ($c = 5\,mM$ in H_2O/MeCN with V:V = 1:1, abcr GmbH). The crystalline powder of D-BiO-NC was dissolved in EtOH (Uvasol), whereas A-BiO-NC was dissolved in DMSO (LC-MS grade, purity by titration: 99.7 %, water content: $\leq 0.1\,\%$, Thermo Scientific, $V = 1.5$ mL) at 80 °C and diluted with MeCN (HiPerSolv Chromanorm, water content: < 30 ppm, $V = 3.5$ mL) to obtain a final concentration of 100 μM, respectively. After passing through a syringe filter (PTFE®, $d = 13$ mm, 0.2 μm), the sample was injected into the ESI source using a Hamilton syringe ($V = 500\,\mu L$) at a flow rate of $180\,\mu L \cdot h^{-1}$. The spray capillary voltage was set to 4.5 kV (positive mode) with a deflection delta of 70.0 V and an end plate offset voltage of 500 V. Mass spectra were processed by smoothing (Savitzky–Golay algorithm, width 0.05 m/z) and isotope patterns were calculated using Bruker Compass DataAnalysis software (Copyright © 2023 Bruker Daltonik GmbH & Co. KG, version 6.1).

2. Cluster Characterization

Nuclear Magnetic Resonance (NMR) Spectroscopy

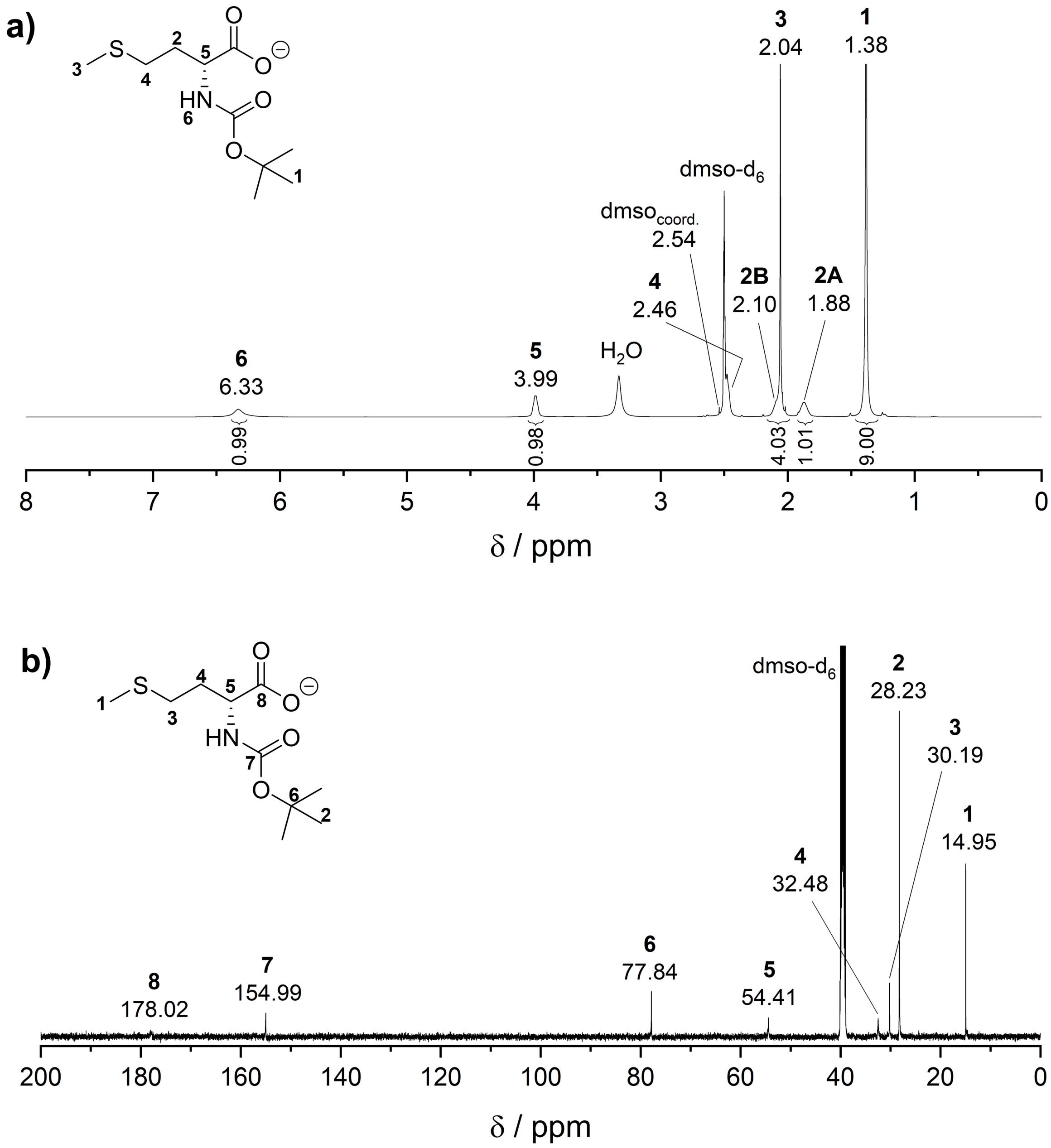

Figure S1: ^{1}H NMR (a) and ^{13}C{^{1}H} NMR spectra (b) of D-BiO-NC in dmso-d_6.

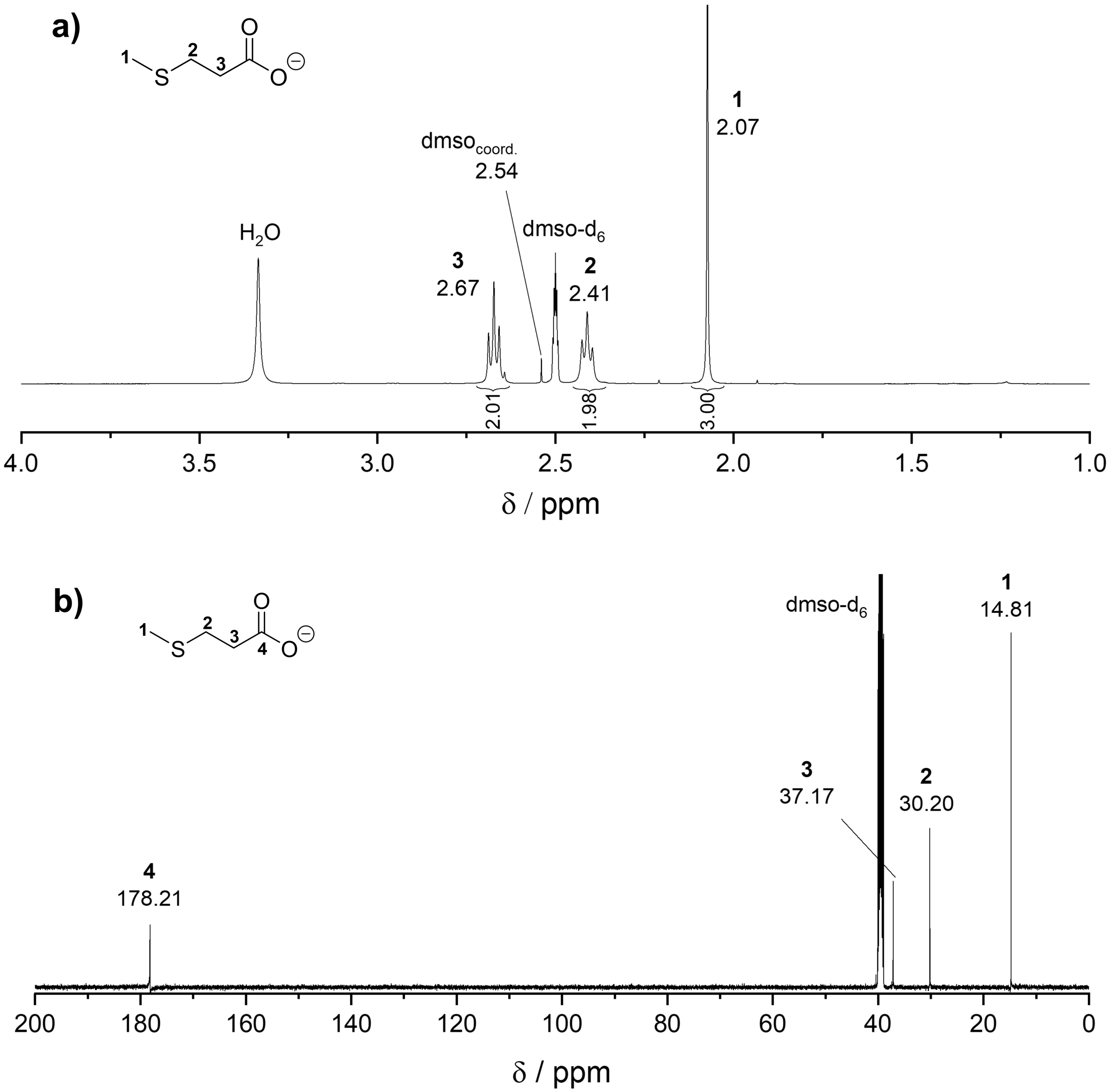

Figure S2: ^1H NMR (a) and $^{13}C\{^1H\}$ NMR spectra (b) of A-BiO-NC in dmso-d_6.

Electrospray Ionization Mass Spectrometry (ESI-MS)

ESI mass spectrometry was used to characterize the molecular composition of D-BiO-NC. Ionization from ethanol (EtOH) generated a distribution of bismuth oxido cluster cations carrying two to three positive charges, with no evidence for additional solvent coordination. The assigned cationic species together with their corresponding m/z values are compiled in

Tab. S1. The survey spectrum in Fig. S3 shows the regions containing doubly and triply charged cations, together with an enlarged view of the trivalent species including assignment of the observed signals. The corresponding calculated isotope patterns of the cluster ions are shown in Fig. S4 for comparison with the experimental data.

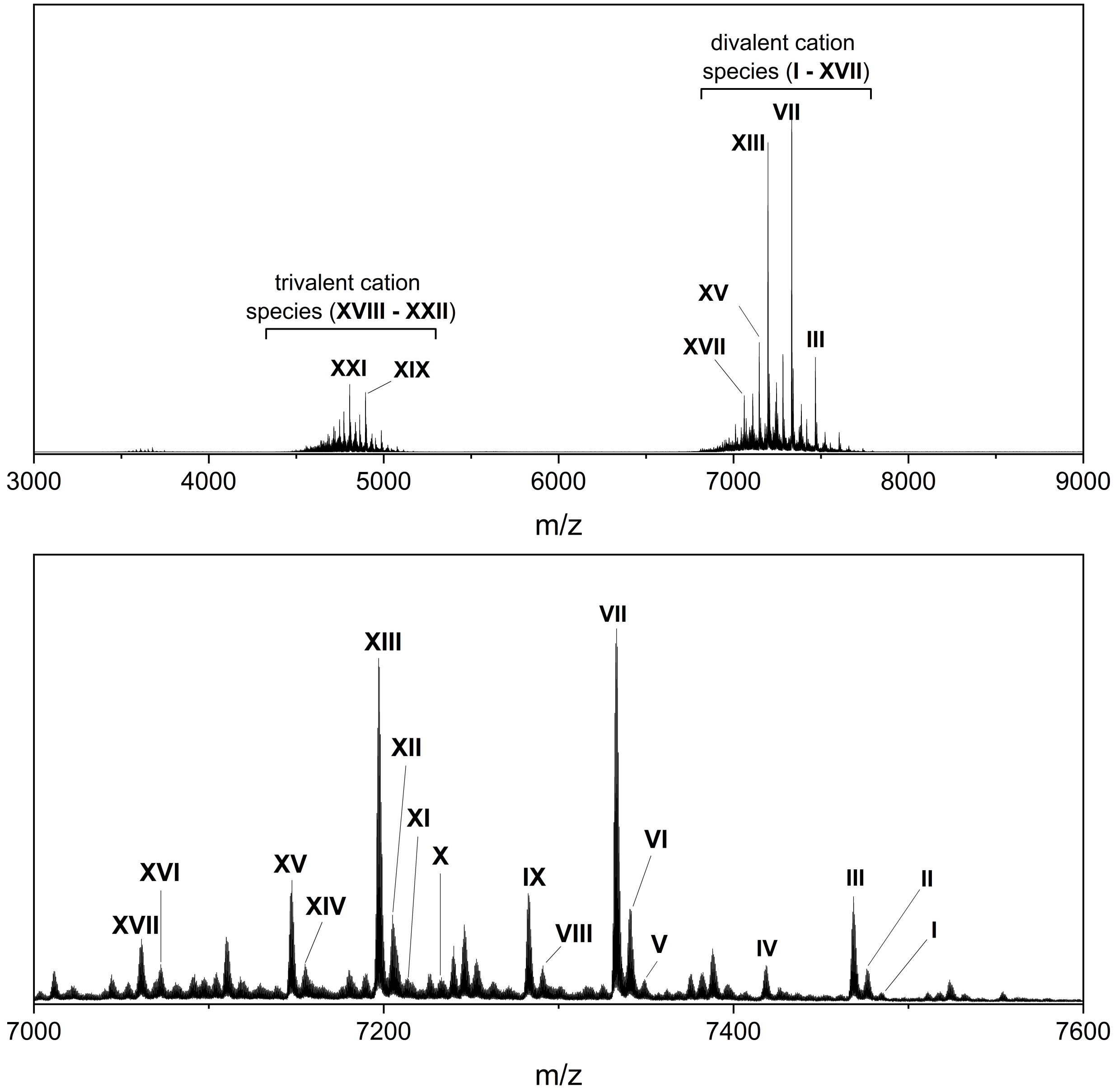

Figure S3: Top: Survey mass spectrum of D-BiO-NC electrosprayed from EtOH showing typical doubly (**I–XVII**) and triply (**XVIII–XXII**) positively charged bismuth oxido nanocluster cations (Tab. S1). Bottom: Cutout of the ESI mass spectrum (m/z = 7000–7800) of D-BiO-NC showing signals assigned to doubly positively charged sodium-adduct species such as $[Bi_{38}O_{45}(\text{Boc-D-Met–O})_{24}Na_2]^{2+}$ (**VII**, m/z = 7333.1259) or $[Bi_{38}O_{45}(\text{Boc-D-Met–O})_{23}Na]^{2+}$ (**XIII**, m/z = 7197.0811), and partially Boc-deprotected fragments such as $[Bi_{38}O_{45}(\text{Boc-D-Met–O})_{23}(\text{D-Met–O})Na_2]^{2+}$ (**IX**, m/z = 7283.0945).

Table S1: Calculated and experimental m/z values of detected cations for D-BiO-NC.

Label	Cation	m/z	
		calcd.	exp.
I	$[Bi_{38}O_{45}(C_{10}H_{18}NO_4S)_{23}(C_{10}H_{18}NO_5S)_2Na_3]^{2+}$	7484.6909	7484.6409
II	$[Bi_{38}O_{45}(C_{10}H_{18}NO_4S)_{24}(C_{10}H_{18}NO_5S)Na_3]^{2+}$	7476.6934	7476.6500
III	$[Bi_{38}O_{45}(C_{10}H_{18}NO_4S)_{25}Na_3]^{2+}$	7468.6960	7468.6632
IV	$[Bi_{38}O_{45}(C_{10}H_{18}NO_4S)_{24}(C_5H_{10}NO_2S)Na_3]^{2+}$	7418.6697	7418.6363
V	$[Bi_{38}O_{45}(C_{10}H_{18}NO_4S)_{22}(C_{10}H_{18}NO_5S)_2Na_2]^{2+}$	7349.1481	7349.1300
VI	$[Bi_{38}O_{45}(C_{10}H_{18}NO_4S)_{23}(C_{10}H_{18}NO_5S)Na_2]^{2+}$	7341.1507	7341.1108
VII	$[Bi_{38}O_{45}(C_{10}H_{18}NO_4S)_{24}Na_2]^{2+}$	7333.1532	7333.1259
VIII	$[Bi_{38}O_{45}(C_{10}H_{18}NO_4S)_{23}(C_5H_{10}NO_2SO)Na_2]^{2+}$	7291.1244	7291.0833
IX	$[Bi_{38}O_{45}(C_{10}H_{18}NO_4S)_{23}(C_5H_{10}NO_2S)Na_2]^{2+}$	7283.1269	7283.0945
X	$[Bi_{38}O_{45}(C_{10}H_{18}NO_4S)_{22}(C_5H_{10}NO_2S)_2Na_2]^{2+}$	7233.1006	7233.0681
XI	$[Bi_{38}O_{45}(C_{10}H_{18}NO_4S)_{21}(C_{10}H_{18}NO_5S)_2Na]^{2+}$	7213.6053	7213.5763
XII	$[Bi_{38}O_{45}(C_{10}H_{18}NO_4S)_{22}(C_{10}H_{18}NO_5S)Na]^{2+}$	7205.1077	7205.0711
XIII	$[Bi_{38}O_{45}(C_{10}H_{18}NO_4S)_{23}Na]^{2+}$	7197.1102	7197.0811
XIV	$[Bi_{38}O_{45}(C_{10}H_{18}NO_4S)_{22}(C_5H_{10}NO_2SO)Na]^{2+}$	7155.5816	7155.5483
XV	$[Bi_{38}O_{45}(C_{10}H_{18}NO_4S)_{22}(C_5H_{10}NO_2S)Na]^{2+}$	7147.0839	7147.0556
XVI	$[Bi_{38}O_{45}(C_{10}H_{18}NO_4S)_{21}(C_{10}H_{18}NO_5S)]^{2+}$	7069.5649	7069.5206
XVII	$[Bi_{38}O_{45}(C_{10}H_{18}NO_4S)_{22}]^{2+}$	7061.5674	7061.5325
XVIII	$[Bi_{38}O_{45}(C_{10}H_{18}NO_4S)_{23}(C_{10}H_{18}NO_5S)Na_3]^{3+}$	4901.7635	4901.7481
XIX	$[Bi_{38}O_{45}(C_{10}H_{18}NO_4S)_{24}Na_3]^{3+}$	4896.4319	4896.4176
XX	$[Bi_{38}O_{45}(C_{10}H_{18}NO_4S)_{22}(C_{10}H_{18}NO_5S)Na_2]^{3+}$	4811.4017	4811.3838
XXI	$[Bi_{38}O_{45}(C_{10}H_{18}NO_4S)_{23}Na_2]^{3+}$	4805.7365	4805.7228
XXII	$[Bi_{38}O_{45}(C_{10}H_{18}NO_4S)_{22}Na]^{3+}$	4715.3747	4715.3476

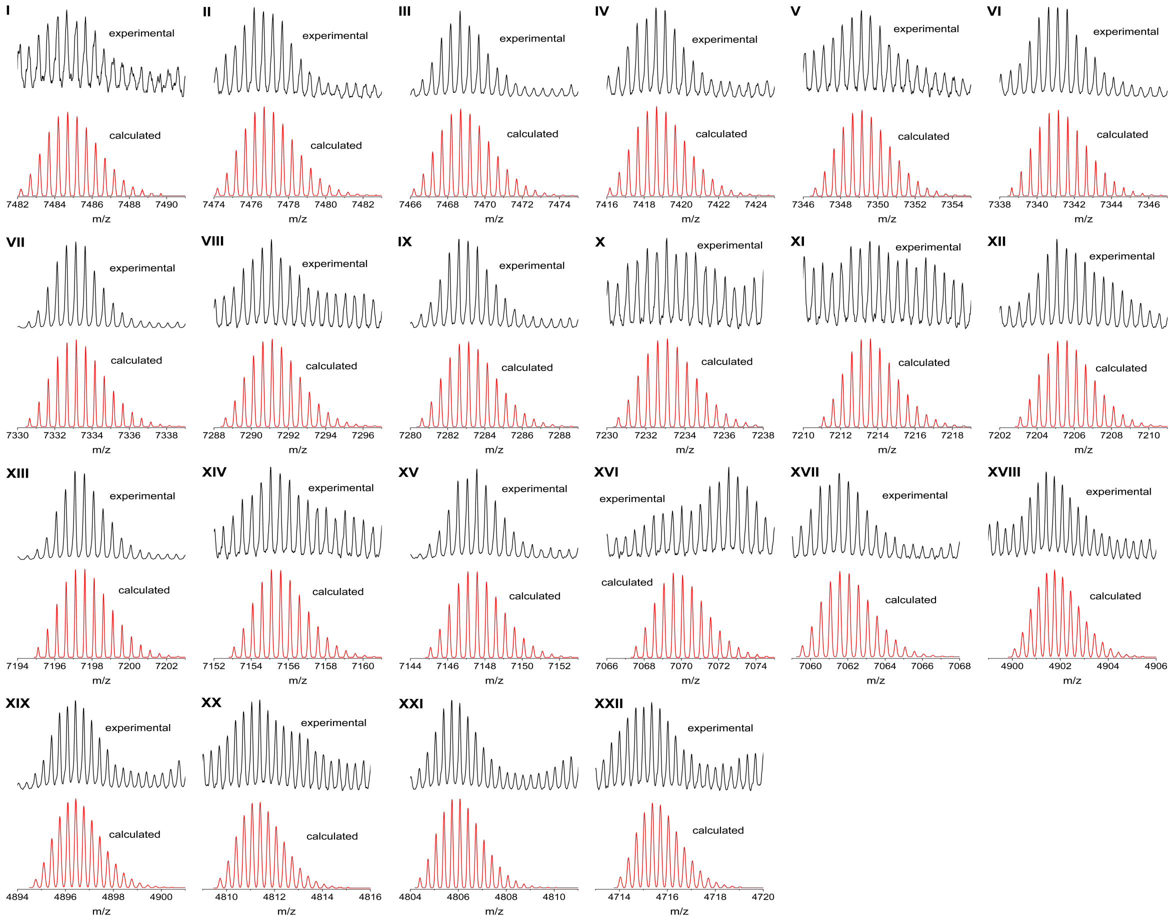

Figure S4: Isotopic patterns (exp. and calcd.) of different doubly and triply positive charged BiO-NC species (**I–XXII**) detected in the gas phase generated from D-BiO-NC after electrospraying from EtOH.

The observed signal sequence can be attributed to the stepwise oxidation of thioether ligands during electrospray ionization. Such mass shifts are consistent with the formation of sulfoxide species at the ligand's sulfur atom, a known electrospray-induced oxidation pathway for thioethers.[3] In the spectra, this oxidation is observed repeatedly observed for several ligands, with up to three consecutive oxygen additions, indicating that multiple ligands within a cluster cation can be oxidized under the ESI-MS conditions. Comparable stepwise sulfur oxidation processes have been described for methionine-containing compounds, in which thioether groups are initially converted to sulfoxides and subsequently to more highly oxidized sulfur species during ESI-MS.[3]

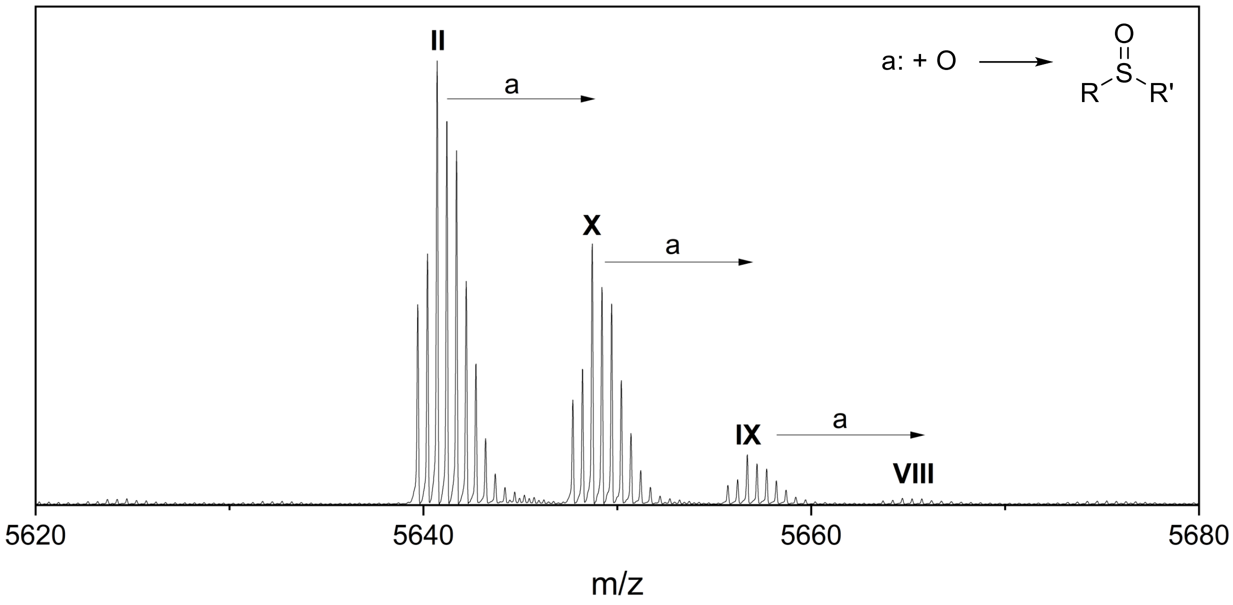

Figure S5: Stepwise oxidation of thioether ligands during electrospray ionization of A-BiO-NC with each successive step corresponding to oxidation of one additional ligand.

Table S2: Calculated and experimental m/z values of detected cations for A-BiO-NC.

Label	Cation	m/z	
		calcd.	exp.
I	$[Bi_{38}O_{45}(O_2C_4H_7S)_{21}]^{3+}$	3720.7917	3720.7923
II	$[Bi_{38}O_{45}(O_2C_4H_7S)_{22}]^{2+}$	5640.6962	5640.7070
III	$[(Bi_{38}O_{45}(O_2C_4H_7S)_{24})(Bi_{38}O_{45}(O_2C_4H_7S)_{21})]^{3+}$	7561.2675	7561.2821
IV	$[(Bi_{38}O_{45}(O_2C_4H_7S)_{21}(O_3C_4H_7S)_3)(Bi_{38}O_{45}(O_2C_4H_7S)_{21})]^{3+}$	7577.2624	7577.2787
V	$[(Bi_{38}O_{45}(O_2C_4H_7S)_{22}(O_3C_4H_7S)_2)(Bi_{38}O_{45}(O_2C_4H_7S)_{21})]^{3+}$	7571.9307	7571.9445
VI	$[(Bi_{38}O_{45}(O_2C_4H_7S)_{23}(O_3C_4H_7S))(Bi_{38}O_{45}(O_2C_4H_7S)_{21})]^{3+}$	7566.5991	7566.6155
VII	$[Bi_{38}O_{45}(O_2C_4H_7S)_{23}Na]^{2+}$	5711.6995	5711.7161
VIII	$[Bi_{38}O_{45}(O_2C_4H_7S)_{19}(O_3C_4H_7S)_3]^{2+}$	5664.6886	5664.7003
IX	$[Bi_{38}O_{45}(O_2C_4H_7S)_{20}(O_3C_4H_7S)_2]^{2+}$	5656.6911	5656.7012
X	$[Bi_{38}O_{45}(O_2C_4H_7S)_{21}(O_3C_4H_7S)]^{2+}$	5648.6936	5648.7049
XI	$[Bi_{38}O_{45}(O_2C_4H_7S)_{18}(O_3C_4H_7S)_3]^{3+}$	3736.7866	3736.7886
XII	$[Bi_{38}O_{45}(O_2C_4H_7S)_{19}(O_3C_4H_7S)_2]^{3+}$	3731.4550	3731.4569
XIII	$[Bi_{38}O_{45}(O_2C_4H_7S)_{20}(O_3C_4H_7S)]^{3+}$	3726.1233	3726.1255

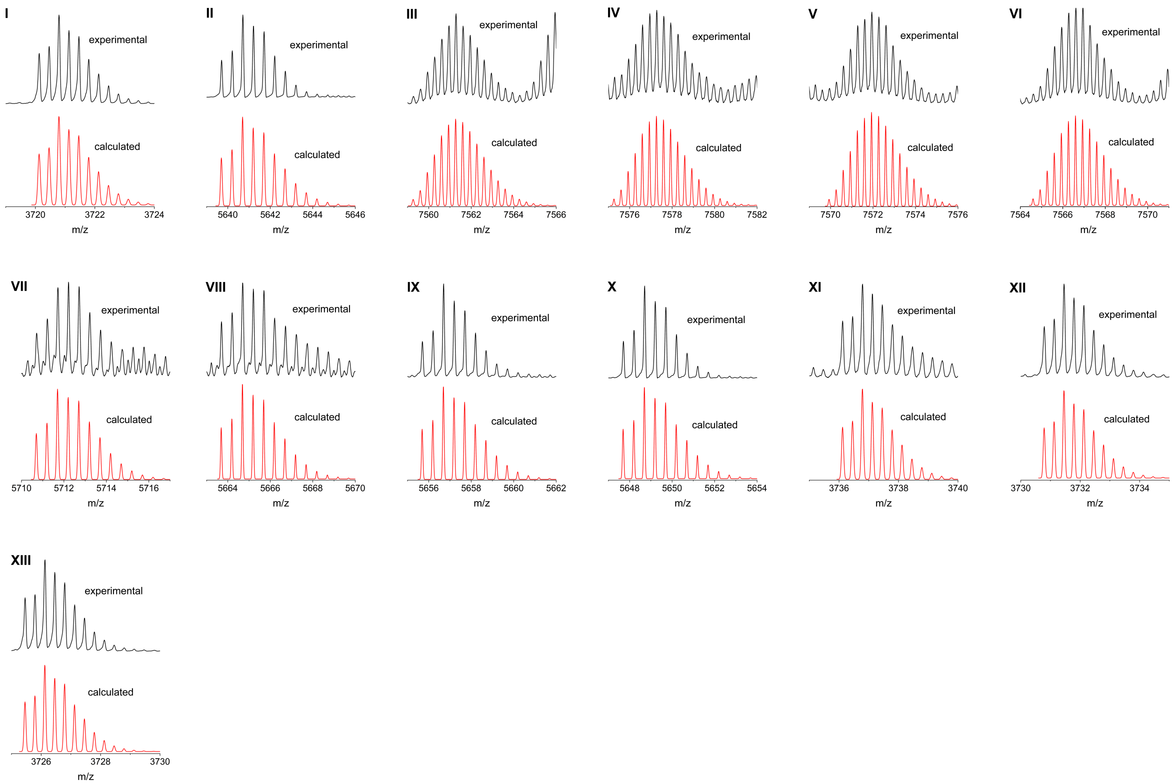

Figure S6: Isotopic patterns (exp. and calcd.) of different doubly and triply positive charged BiO-NC species (**I–XIII**) detected in the gas phase generated from A-BiO-NC after electrospraying from dmso.

Attenuated Total Reflectance Infrared (ATR-IR) Spectroscopy

The ATR-IR measurements (Fig. S7 and Fig. S8) of sodium salts C, D and nanoclusters D-BiO-NC and A-BiO-NC reveal very similar spectra, showing all expected vibrational bands of the sodium salts. Only minor shifts in peak positions and broader signals are observed for nanoclusters D-BiO-NC and A-BiO-NC, which can be attributed to the different coordination environments in the clusters compared to their sodium salts. In addition, almost all spectra exhibit a broad signal in the range of 3100–3600 cm^{-1}, which can be assigned to NH and/or OH vibrations. The OH vibrations are most likely due to residual water as a result of the reaction or washing step. As expected, the IR spectra of nanoclusters D-BiO-NC and A-BiO-NC show the absence of nitrate bands (monodentate coordinating nitrate ($\nu_{as}(NO_2)$: 1432 cm^{-1}, $\nu_s(NO_2)$: 1000 cm^{-1}); bidentate coordinating nitrate ($\nu_{as}(NO_2)$: 1382 cm^{-1}, $\nu_s(NO_2)$:

1266 cm^{-1}); and non-coordinating nitrate (ν(N=O): 1741 cm^{-1}, 1640 cm^{-1})) in comparison to the starting material A, which indicates the complete substitution of the nitrate ligands.[2] The most pronounced difference in the spectra of D-BiO-NC and A-BiO-NC is a broad Bi–O vibration of the Bi–O framework at around 460–600 cm^{-1}. Furthermore the IR spectra of L-BiO-NC and D-BiO-NC show identical vibrational features, which indicates that they have equivalent ligand coordination and chemical compositions as expected for enantiomers. Circular dichroism measurements reveal mirror-image Cotton effects, confirming opposite handedness and establishing the nanoclusters as an enantiomeric pair.

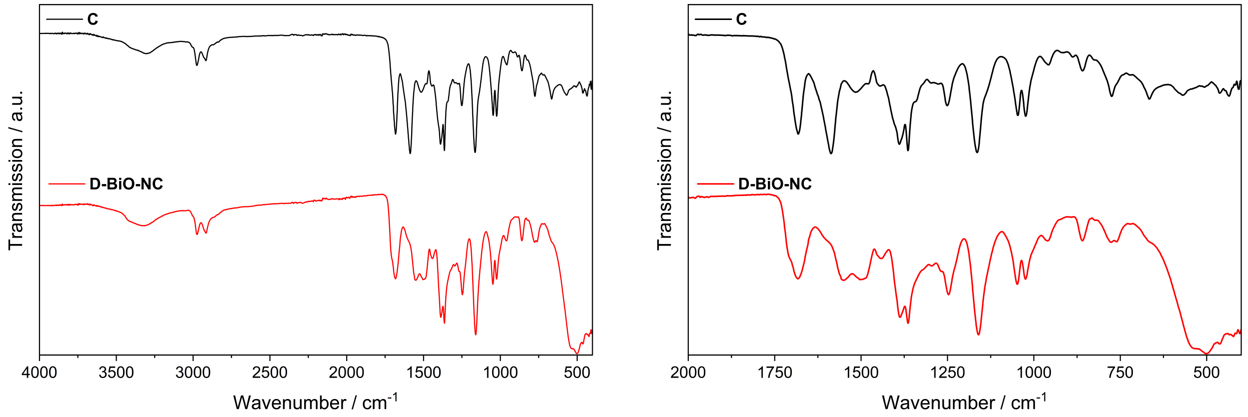

Figure S7: Comparison of the ATR-IR spectra left (full spectra) and right (cut-out) of the sodium salt C (black) and nanocluster D-BiO-NC (red).

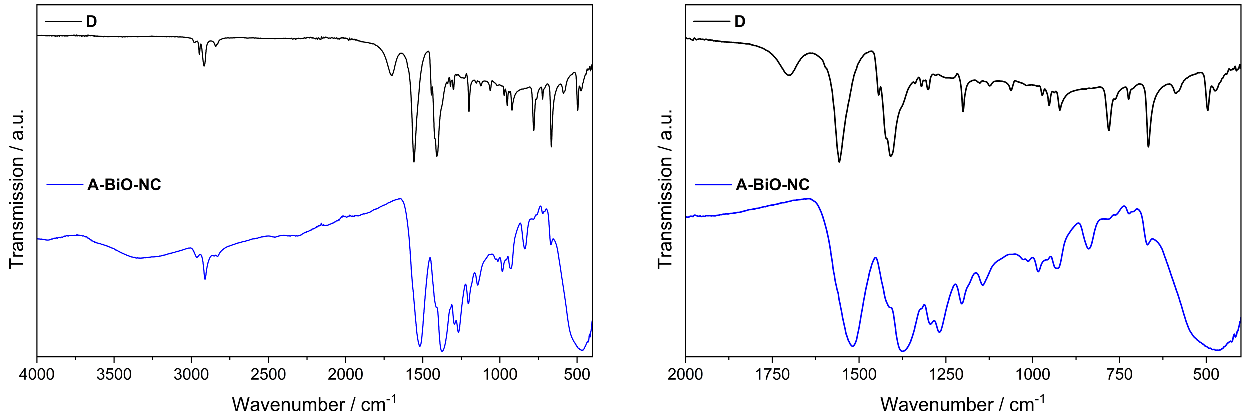

Figure S8: Comparison of the ATR-IR spectra left (full spectra) and right (cut-out) of the sodium salt D (black) and nanocluster A-BiO-NC (blue).

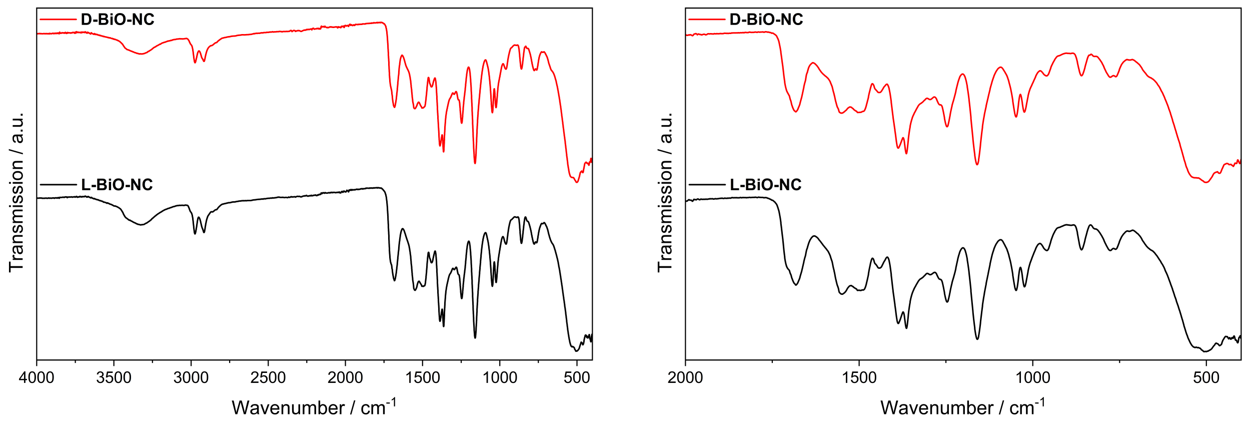

Figure S9: Comparison of the ATR-IR spectra left (full spectra) and right (cut-out) of L-BiO-NC (black) and D-BiO-NC (red).

Circular Dichroism (CD) Spectroscopy

The CD spectrum of A-BiO-NC, measured in chloroform ($c = 3 \cdot 10^{-5}\,\mathrm{mol\,L^{-1}}$) shows no detectable signal in the range of 250-300 nm, consistent with the achiral character of the compound.

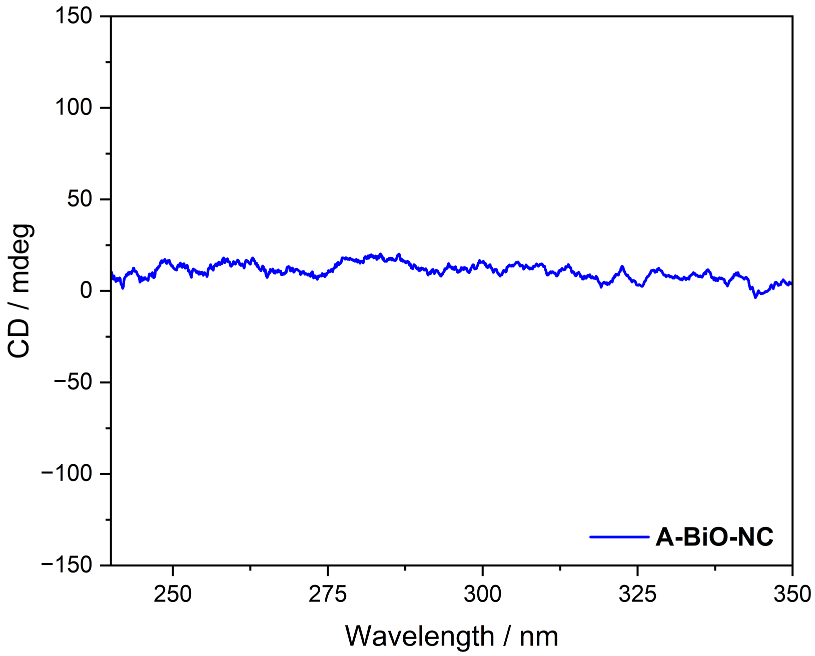

Figure S10: CD spectra of the A-BiO-NC ($c = 3 \cdot 10^{-5}\,\mathrm{mol\,L^{-1}}$) in chloroform.

Powder X-Ray Diffraction (PXRD) and Dynamic Light Scattering (DLS)

Fig. S11 summarizes the PXRD patterns of the as-prepared D-BiO-NC and A-BiO-NC. Both nanoclusters show characteristic diffraction features typical for this cluster family. In particular, the pronounced reflection observed in the 2θ range of 4–6° confirms the preservation of the intact $\{Bi_{38}O_{45}\}$ core structure. The corresponding interlayer distances determined for D-BiO-NC is d = 1.90 nm ($2\theta = 4.64°$), in good agreement with the values reported previously for L-BiO-NC,[2] whereas A-BiO-NC shows a slightly smaller distance of d = 1.83 nm (2θ = 4.82°). These values support the increase in overall molecular dimensions upon ligand exchange in comparison to the starting material A with d = 1.69 nm ($2\theta = 5.23°$).[2] The calculated interlayer distances of the BiO-NCs are in good agreement with a growing radius due to the larger ligands used.

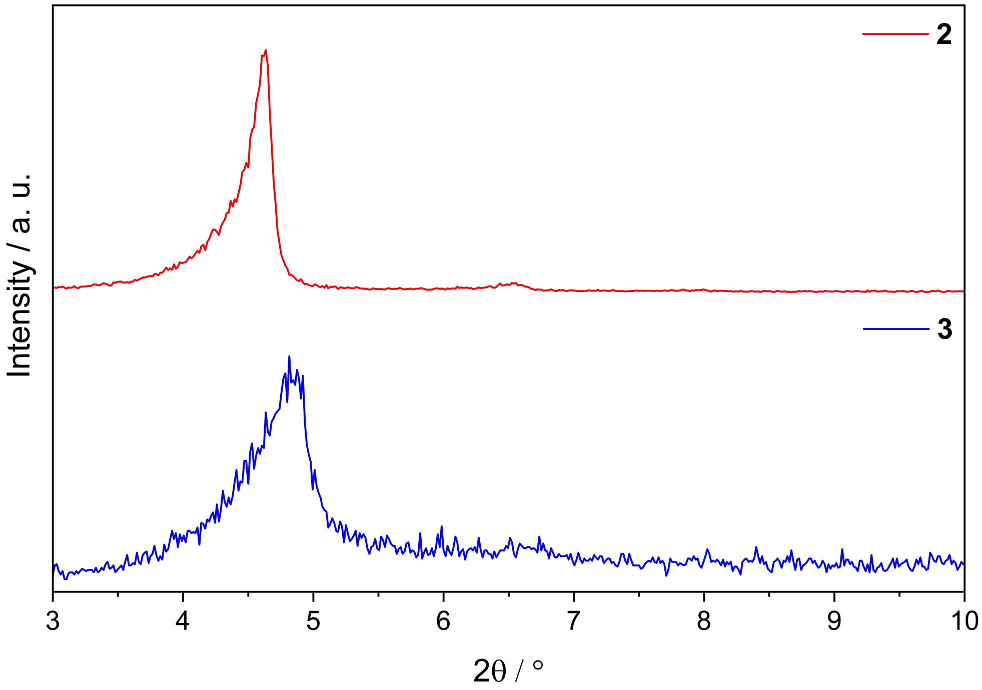

Figure S11: Comparison of the PXRD patterns of D-BiO-NC and A-BiO-NC.

The size determination of the NCs by DLS can also depend on the choice of solvent. Due to solubility differences, D-BiO-NC and L-BiO-NC were dissolved in ethanol, while A-

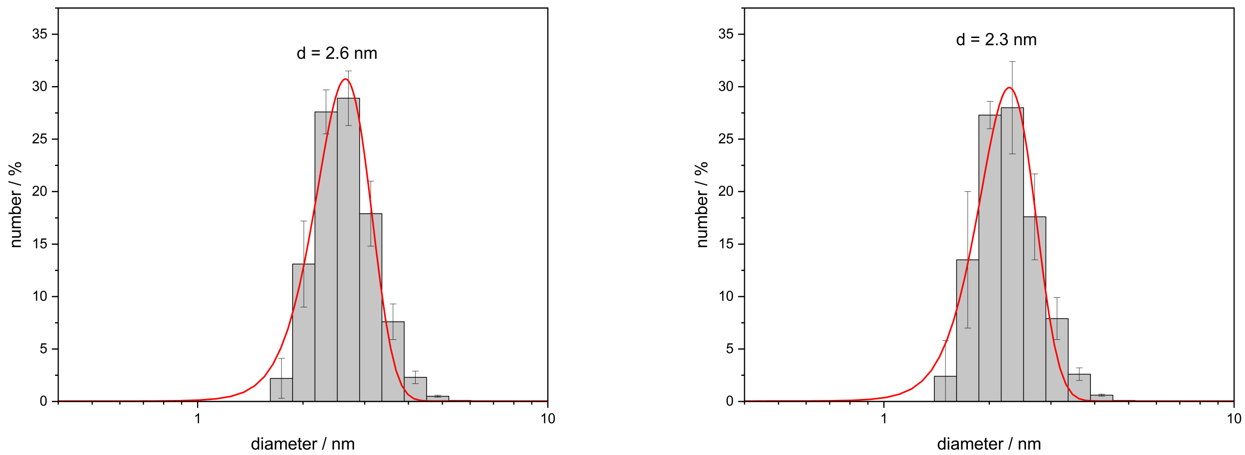

Figure S12: Particle size distributions of D-BiO-NCs in ethanol and DMSO determined by dynamic light scattering (DLS).

BiO-NC was dissolved in DMSO and deposited onto the substrates via drop casting from DMSO/EtOH solution. Using D-BiO-NC as an example, DLS measurements were performed in both ethanol and DMSO, as shown in Fig. S12. For D-BiO-NC, the hydrodynamic diameter was determined to be approximately 2.6 nm in ethanol and 2.3 nm in DMSO, which is in the same range as observed for its corresponding enantiomer L-BiO-NCs.[2]

Ultraviolet-Visible (UV-vis) Spectroscopy

We further investigated the electronic properties using UV–vis absorption spectroscopy, as shown in Fig. S13. The optical band gap of the A-BiO-NC is approximately 3.5 eV and thus similar to that of the chiral BiO-NCs (3.6 eV).[4]

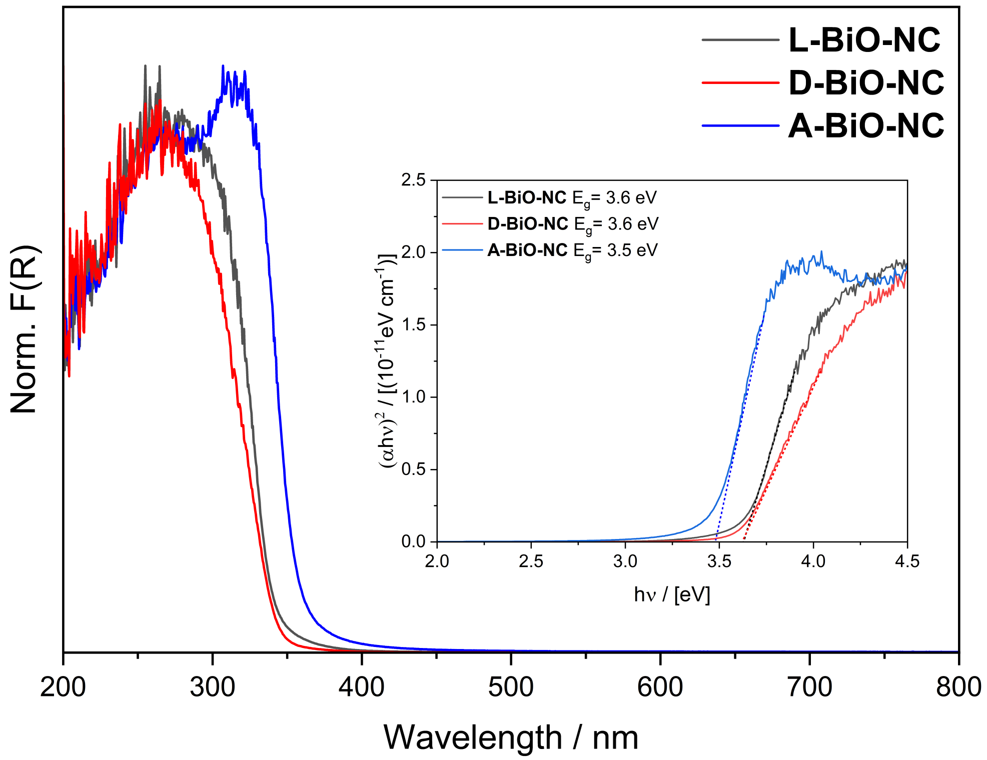

Figure S13: UV-vis spectra in diffuse reflection of L-BiO-NC, D-BiO-NC and A-BiO-NC.

3. Scanning transmission electron microscopy (STEM)

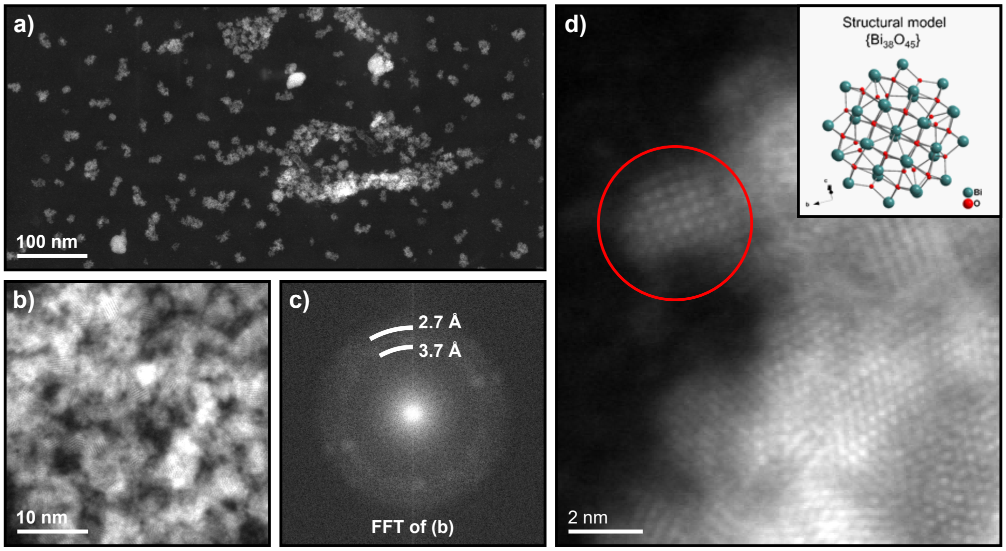

Figure S14: STEM-HAADF data of L-BiO-NCs. (a) Low-magnification STEM image showing agglomerations of L-BiO-NCs. Note: The agglomeration occurred in the highly concentrated solution prior to sample deposition. (b) Magnified view of a BiO-NC ensemble. (c) FFT of the cluster ensemble shown in (b). (d) Single BiO-NC highlighted by the circle. Inset: Structural model of a Bi38O45 cluster. For clarity, the ligands are omitted.

In addition, we acquired high-angle annular dark-field scanning transmission electron microscopy (HAADF-STEM) images of the L-BiO-NCs, as shown in Fig. S14. The images were recorded using a 300 keV Spectra microscope equipped with HAADF detectors. The BiO-NCs were deposited from solution onto a thin carbon support film by drop-casting and subsequently transferred to a TEM grid. For the STEM measurements, an aged BiO-NC solution was used. As a result, the clusters had already begun to aggregate in solution, forming cluster assemblies that are clearly visible in the low-magnification image in panel (a). Nevertheless, intact individual clusters can still be clearly identified in the higher-magnification images.

Based on total scattering measurements in solution, Anker et al.[5] demonstrated by pair distribution function (PDF) analysis that functionalized [Bi38O45(L)24] (L = $NO3^-$ and OMc^-) nanoclusters exhibit characteristic Bi–Bi distances of approximately 3.8 Å. In excellent agreement, the FFT of the HAADF-STEM image shown in Fig. S14(c) reveals a

characteristic periodicity of 3.7 Å. Furthermore, this value agrees well with the Bi–Bi distances (3.7–3.9 Å) determined from the single-crystal structure of methacrylate-substituted $Bi_{38}O_{45}$ nanoclusters (see inset in Fig. S14(d); the methacrylate ligands (OMc^-) are omitted for clarity).[1,4,6] Closer inspection of the FFT also reveals a second, weaker intensity ring corresponding to a spacing of approximately 2.7 Å, consistent with the expected average Bi–O distances.

Together, these results indicate that the Bi38O45 cluster core remains structurally intact upon deposition.

4. STM data and IV-spectroscopy

In the main text we showed the apparent height profile for L-Bio-NC. In Fig. S15 we provide in addition also the height profiles for D-BiO and A-BiO. The average cluster sizes for the chiral BiO-NCs shown in panels a) and b) and the A-BiO-NC in panel c) are 2.5 (±0.2) nm and 1.9 (±0.2) nm, respectively. The values correlate nicely with the diameters determined by DLS.

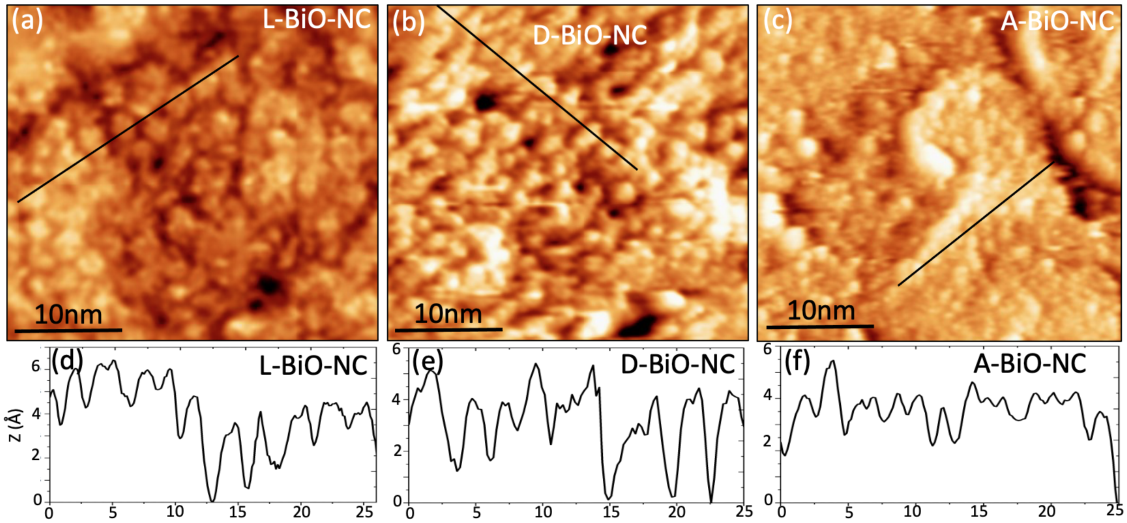

Figure S15: (a-c) STM images of L-BiO, D-BiO and A-BiO nanoclusters, respectively, and the corresponding apparent height profiles (d-f).

The STS data for the various BiO-NCs on Au/Co/Au substrates with different magneti-

zation states were measured at least ten times and subsequently averaged. The corresponding data dispersion is shown in Figure S16.

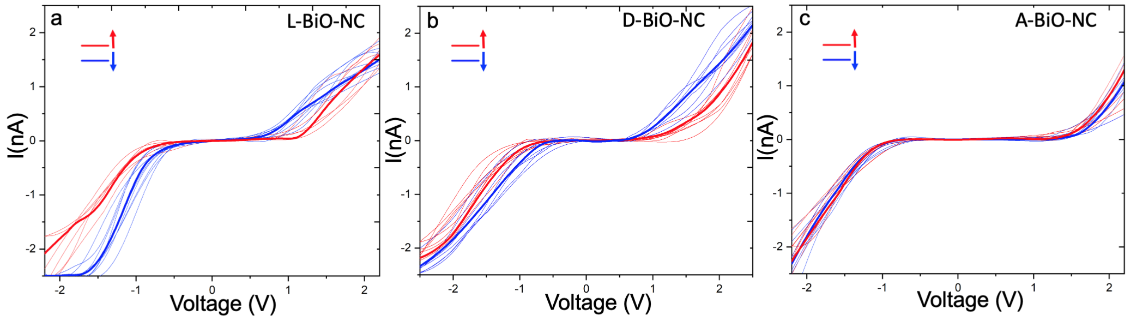

Figure S16: CISS I–V curves used in this manuscript. The thin lines show individual measurements, while the solid lines correspond to the averaged curves shown in the main text.

The different linker groups used for the A-BiO-NC are associated with different solubilities. While the chiral clusters were dissolved in ethanol, the achiral cluster was best dissolved in DMSO. The data are shown in the main text and in Fig. S16. To rule out that the vanishing CISS effect for the A-BiO-NC is caused by the different solvent, we also performed CISS measurements using a mixture of DMSO and ethanol. As shown in Fig. S17, ethanol has no influence on any potentially solvent-induced chirality.

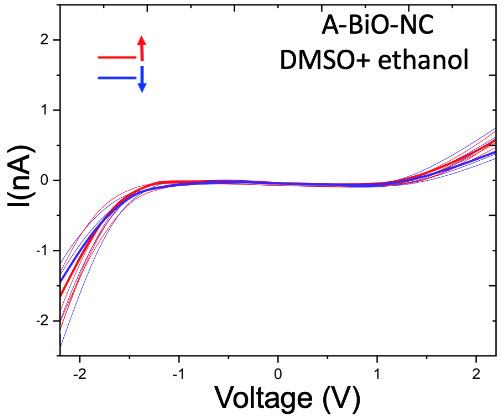

Figure S17: Averaged CISS I–V curve for A-BiO-NC in DMSO+ethanol solvent showing also no CISS signal like the spectra in Fig. S16c).

References

1. Miersch, L.; Schlesinger, M.; Troff, R. W.; Schalley, C. A.; Rüffer, T.; Lang, H.; Zahn, D.; Mehring, M. Hydrolysis of a Basic Bismuth Nitrate—Formation and Stability of Novel Bismuth Oxido Clusters. *Chemistry – A European Journal* **2011**, *17*, 6985–6990.

2. Morgenstern, A.; Thomas, R.; Selyshchev, O.; Weber, M.; Tegenkamp, C.; Zahn, D. R. T.; Mehring, M.; Salvan, G. Anchoring Atomically Precise Chiral Bismuth Oxido Nanoclusters on Gold: The Role of Amino Acid Linkers. *Langmuir* **2024**, *40*, 16320–16329.

3. Chen, M.; Cook, K. D. Oxidation Artifacts in the Electrospray Mass Spectrometry of Aβ Peptide. *Analytical Chemistry* **2007**, *79*, 2031–2036, PMID: 17249640.

4. Thomas, R.; Nguyen, T. N. H.; Weber, M.; Rüffer, T.; Göhler, F.; Deka, A.; Pöppl, A.; Seyller, T.; Tegenkamp, C.; Mehring, M. Atomically precise bismuth oxido nanoclusters: cerium doping for optical modification and supramolecular self-assembly on Au(111). *Nanoscale* **2025**, *17*, 18291–18304.

5. Anker, A. S.; Christiansen, T. L.; Weber, M.; Schmiele, M.; Brok, E.; Kjær, E. T. S.; Juhás, P.; Thomas, R.; Mehring, M.; Jensen, K. M. Structural Changes during the Growth of Atomically Precise Metal Oxido Nanoclusters from Combined Pair Distribution Function and Small-Angle X-ray Scattering Analysis. *Angew. Chem. Int. Ed.* **2021**, *60*, 20407–20416.

6. Thomas, R.; Kumar Kuppusamy, S.; Rüffer, T.; Weber, M.; Ruben, M.; Mehring, M. Europium Doped Atomically Precise Bismuth Oxido Nanoclusters as Molecular Building Blocks for Photoluminescent Hybrid Materials. *ChemRxiv* **2026**, https://doi.org/10.26434/chemrxiv.15000367/v1.
